# Untargeted metabolomic analysis in cats with naturally occurring inflammatory bowel disease and alimentary small cell lymphoma

**DOI:** 10.1038/s41598-021-88707-5

**Published:** 2021-04-28

**Authors:** Sina Marsilio, Betty Chow, Steve L. Hill, Mark R. Ackermann, J. Scot Estep, Benjamin Sarawichitr, Rachel Pilla, Jonathan A. Lidbury, Joerg M. Steiner, Jan S. Suchodolski

**Affiliations:** 1 Department of Medicine and Epidemiology, School of Veterinary Medicine, University of California, Davis, One Shields Avenue, Davis, CA 95616 USA; 2 Gastrointestinal Laboratory, Texas A&M University, College Station, TX USA; 3 Veterinary Specialty Hospital, San Diego, CA USA; 4 Oregon Veterinary Diagnostic Laboratory, Carlson College of Veterinary Medicine, Oregon State University, Corvallis, OR USA; 5 Texas Veterinary Pathology, LLC., San Antonio, TX USA; 6 Present Address: VCA Animal Specialty and Emergency Center, Los Angeles, CA USA; 7 Present Address: Flagstaff Veterinary Internal Medicine Consulting, Flagstaff, AZ USA

**Keywords:** Diagnostic markers, Gastrointestinal models, Cancer, Molecular biology

## Abstract

Feline chronic enteropathy (CE) is a common gastrointestinal disorder in cats and mainly comprises inflammatory bowel disease (IBD) and small cell lymphoma (SCL). Differentiation between IBD and SCL can be diagnostically challenging. We characterized the fecal metabolome of 14 healthy cats and 22 cats with naturally occurring CE (11 cats with IBD and 11 cats with SCL). Principal component analysis and heat map analysis showed distinct clustering between cats with CE and healthy controls. Random forest classification revealed good group prediction for healthy cats and cats with CE, with an overall out-of-bag error rate of 16.7%. Univariate analysis indicated that levels of 84 compounds in cats with CE differed from those in healthy cats. Polyunsaturated fatty acids held discriminatory power in differentiating IBD from SCL. Metabolomic profiles of cats with CE resembled those in people with CE with significant alterations of metabolites related to tryptophan, arachidonic acid, and glutathione pathways.

## Introduction

Feline chronic enteropathy (CE) is a spontaneously arising disorder in cats that is especially common in the elderly cat population. It is defined as the chronic presence (i.e., presence for longer than 3 weeks) of signs of gastrointestinal disease such as weight loss, vomiting and/or diarrhea in the absence of infectious intestinal or extraintestinal causes^1^. The most common types of CE in cats are inflammatory bowel disease (IBD) and alimentary small cell lymphoma (SCL)^2–5^. Diagnosis and differentiation require invasive and expensive procedures, including the collection and histopathologic examination of intestinal tissue biopsies. Therapeutic strategies generally consist of treatment with immunosuppressive drugs such as corticosteroids for cases of IBD and or cytotoxic drugs such as chlorambucil for cases with SCL^2,6,7^. Hence, the discovery of less invasive biomarkers for the diagnosis and differentiation of CE in cats and the identification of new therapeutic targets would be highly desirable.

Studies in human subjects have revealed global metabolic changes in people with IBD and the potential to use metabolomic profiling for the diagnosis and differentiation of IBD and IBD subtypes such as Crohn’s disease and ulcerative colitis^8–13^. Common metabolic perturbations described included pathways affecting tryptophan and other amino acids, fatty acids, bile acids, sphingolipids, and biogenic amines^8–13^.

Although metabolomic profiles have been reported in plasma^14,15^ serum^16^ and urine^17^ of healthy cats, information from untargeted metabolomic studies in cats with CE are lacking.

Feline alimentary SCL shows some histological parallels to monomorphic epitheliotropic intestinal T-cell lymphoma (MEITL) in people (i.e., formally known as enteropathy associated T-cell lymphoma Type 2 (EATL Type 2)), such as a monomorphic infiltration of the intestinal mucosa with small to medium lymphocytes of predominantly T-cell origin, epitheliotropism, and a predilection for the small intestine^4,18,19^. However, while there are histopathological parallels, the clinical course of feline SCL and EATL Type 2 is very different. While alimentary SCL in cats is slowly progressing with a median survival time of 1.5 to 3.5 years^20,21^, MEITL in people is a clinically aggressive disease and generally associated with a poor prognosis^18^. Recently, other authors suggested that the cat might be a suitable model for indolent digestive T-cell lymphoproliferative disorder^22,23^. This rare disorder in people is characterized by a superficial monoclonal intestinal T-cell infiltrate and the disease is generally indolent or slowly progressive^19,22,24–27^.

We hypothesized that cats with CE have metabolomic perturbations and that metabolomic profiling can distinguish cats with IBD from cats with SCL. We further hypothesized that metabolic perturbances observed in cats with CE would be similar to those observed in humans with IBD.

## Results

A total of 36 cats were enrolled into this study, 14 healthy cats and 22 cats with chronic enteropathy (11 with IBD and 11 with SCL). A fecal sample was collected from all cats. Demographic characteristics are shown in Table 1.

**Table 1:** Comparison of demographic data between healthy cats and cats with chronic enteropathy (CE).

	Healthy	CE	P value
Number of cats	14	22	
Median age in years (range)	8.5 (3–15)	10.5 (2–16)	0.353
Median BW in kg (range)	5.5 (3.9–8.0)	4.6 (2.5–7.64)	0.007
Median BCS (range)	5.5 (5–9)	4 (1–9)	< 0.001
Sex	18 FS, 20 MN	11 FS, 16 MN	> 0.999
Breeds	13 DSH, 1 mixed breed	6 DSH, 2 DMH, 2 DLH, 1 mixed breed	

*BCS* body condition score, *1–4* underweight, *5* ideal, *6–9* overweight, *BW* body weight, *DLH* domestic longhair, *DMH* domestic medium hair, *DSH* domestic shorthair, *FS* female spayed, *MN* male neutered.

Age (p = 0.353) and sex distribution (p > 0.999) were comparable between control cats and cats with CE (Table 1). Cats with CE had a significantly lower body weight (p = 0.007) and body condition score^28^ (p < 0.001).

Cats with SCL were significantly older (median age: 12 years, range: 9–15 years) than cats with IBD (median age: 7 years, range: 2–16; p = 0.028). Cats with IBD and cats with SCL did not differ significantly regarding sex, body weight, or body condition score (BCS). Cats with CE had a median feline chronic enteropathy activity index (FCEAI)^29^ of 6 (range: 2–11). However, the FCEAI did not differ between cats with IBD (median: 6, range: 3–11) and cats with SCL (median: 5, range: 2–10; p = 0.176).

A total of 856 named metabolites were detected. Nonparametric univariate analysis using a FDR of 5% revealed a total of 84 metabolites that differed significantly between control cats and cats with CE (Table 2).

**Table 2:** Metabolites and pathways significantly altered in cats with chronic enteropathy (CE).

Class/pathway and metabolites		P-value	Q-value	Fold change direction (↑ or ↓) and magnitude
Sub-pathway	Metabolite			
**Amino acids and metabolites**				
Alanine and aspartate metabolism	Alanine	0.0043	0.0470	↑2.2936
	Aspartate	0.0022	0.0346	↑2.7186
Glutathione metabolism	Gamma-glutamylglutamine	0.0014	0.0331	↑5.3251
	2-Hydroxybutyrate/2-hydroxyisobutyrate	0.0004	0.0228	↑9.0087
Glycine, serine and threonine metabolism	2-Methylserine	0.0015	0.0331	↑3.5909
	Glycine	0.0035	0.0445	↑2.2987
Guanidino and acetamido metabolism	Guanidinosuccinate	0.0014	0.0331	↑3.6303
Leucine, Isoleucine and Valine Metabolism	3-Methylglutaconate	0.0022	0.0346	↓0.3917
	Alpha-hydroxyisovalerate	0.0022	0.0346	↑4.804
	Isoleucine	0.0043	0.0470	↑2.2281
	Leucine	0.0028	0.0383	↑2.3409
	Valine	0.0021	0.0347	↑2.9777
Methionine, cysteine, SAM and taurine metabolism	Cysteine sulfinic acid	0.0007	0.0258	↑2.9493
Phenylalanine metabolism	Phenylalanine	0.0039	0.0470	↑2.4088
Tryptophan metabolism	5-Hydroxyindoleacetate	0.0011	0.0327	↓0.4893
	2-Oxindole-3-acetate	< 0.0001	0.0089	↓0.1789
	Indole-3-carboxylic acid	0.0023	0.0347	↑2.1828
Tyrosine metabolism	3-(4-hydroxyphenyl)lactate	0.0025	0.0361	↑19.648
	Gentisate	0.0016	0.0331	↓0.3671
**Carbohydrate**				
Pentose metabolism	Xylose	0.0022	0.0347	↓0.2278
**Cofactors and vitamins**				
Nicotinate and nicotinamide metabolism	N1-Methyl-4-pyridone-3-carboxamide	0.0001	0.0155	↑4.6074
Tocopherol metabolism	Alpha-CEHC	0.0015	0.0331	↑ 7.7239
	Delta-tocopherol	0.0039	0.0470	↑2.3009
Vitamin A metabolism	Beta-cryptoxanthin	0.0003	0.0228	↓0.2144
	Carotene diol (1)	0.0005	0.0228	↓0.3609
	Carotene diol (2)	0.0005	0.0228	↓0.2826
	Carotene diol (3)	0.0003	0.0228	↓0.2709
**Lipids**				
Ceramide	Ceramide (d16:1/24:1, d18:1/22:1)	0.0045	0.0490	↑8.3153
	Ceramide (d18:1/14:0, d16:1/16:0)	0.0013	0.0331	↑5.2335
	N-palmitoyl-sphingadienine (d18:2/16:0)	0.0023	0.0347	↑5.5645
	N-palmitoyl-sphingosine (d18:1/16:0)	0.0043	0.0470	↑3.3205
Fatty acid, hydroxyl	LAHSA (18:2/OH-18:0)	0.0003	0.0228	↑35.365
	OAHSA (18:1/OH-18:0)	0.0002	0.0228	↑37.144
	PAHSA (16:0/OH-18:0)	0.0005	0.0228	↑32.316
Fatty acid, branched	12-Methyltridecanoate (i14:0)	0.0005	0.0228	↓0.3418
Fatty acid, dicarboxylate	Adipate (C6-DC)	0.0017	0.0341	↑2.7411
	Azelate (C9-DC)	0.0014	0.0331	↓0.3523
	Dodecenedioate (C12:1-DC)	0.0031	0.0413	↑2.2159
	Octadecenedioate (C18:1-DC)	0.0005	0.0228	↑3.3075
	Pimelate (C7-DC)	0.0043	0.0470	↓0.46686
	Sebacate (C10-DC)	0.001	0.0327	↓0.3776
Fatty acid, monohydroxy	3-Hydroxyoleate	0.0016	0.0331	↑4.8978
	3-Hydroxysuberate	0.0053	0.0511	↑1.7385
	10-Hydroxystearate	< 0.0001	0.0149	↑5.766
	13-HODE + 9-HODE	0.0011	0.0327	↑2.0752
Fatty acid metabolism(acyl carnitine)	Oleoylcarnitine (C18:1)	0.0025	0.0361	↑4.2787
	Palmitoylcarnitine (C16)	0.0042	0.0470	↑4.6424
Long chain fatty acid	10-Heptadecenoate (17:1n7)	0.0043	0.0470	↑5.9203
	10-Nonadecenoate (19:1n9)	0.0025	0.0361	↑11.735
	Eicosenoate (20:1)	0.0008	0.0287	↑12.345
	Erucate (22:1n9)	0.0013	0.0331	↑12.773
	Myristate (14:0)	0.0011	0.0327	↑5.9909
	Oleate/vaccenate (18:1)	0.0031	0.0413	↑5.6274
Lysophospholipid	1-Stearoyl-GPC (18:0)	0.0035	0.0445	↑13.363
Lysoplasmalogen	1-(1-enyl-oleoyl)-GPE (P-18:1)	0.0007	0.0258	↑7.8633
Medium chain fatty acids	Laurate (12:0)	0.0005	0.0228	↑11.522
Polyunsaturated fatty acids (n3 and n6)	Arachidonate (20:4n6)	0.0025	0.0361	↑7.9203
	Docosadienoate (22:2n6)	0.0018	0.0345	↑8.8555
	Docosahexaenoate (DHA; 22:6n3)	0.0048	0.0499	↑18.558
	Docosatrienoate (22:3n3)	0.0028	0.0383	↑7.0842
	Heneicosapentaenoate (21:5n3)	0.0023	0.0347	↑12.74
	Hexadecatrienoate (16:3n3)	0.0028	0.0383	↑6.5171
	Nisinate (24:6n3)	0.0011	0.0327	↑10.845
Sphingomyelins	Behenoyl sphingomyelin (d18:1/22:0)	0.0040	0.0470	↑24.455
	Sphingomyelin (d17:1/16:0, d18:1/15:0, d16:1/17:0)	0.0001	0.0155	↑9.9925
	Sphingomyelin (d18:1/14:0, d16:1/16:0)	0.0004	0.0228	↑10.726
	Sphingomyelin (d18:2/16:0, d18:1/16:1)	0.0040	0.0470	↑4.4038
Sphingosines	Hexadecasphingosine (d16:1)	0.0048	0.0499	↑3.4953
Sterols	Beta-sitosterol	0.0005	0.0228	↓0.3931
	Campesterol	0.0039	0.0470	↑3.1733
	Ergosterol	0.0016	0.0331	↓0.357
	Fucosterol	0.0020	0.0347	↓0.3795
	Stigmasterol	< 0.0001	0.0089	↓0.3387
**Nucleotide**				
Pyrimidine metabolism, cytidine containing	5-Hydroxymethylcytosine	0.0019	0.0346	↑7.6263
Pyrimidine metabolism, thymine containing	Thymine	0.0016	0.0331	↑2.2599
**Xenobiotics**				
Benzoate metabolism	3-(3-Hydroxyphenyl)propionate	< 0.0001	0.0089	↓0.1316
Chemical	4-Acetamidobenzoate	0.0004	0.0228	↓0.2582
Drug-topical agents	Salicylate	0.001	0.0327	↓0.3601
Food component/plant	3-Hydroxycinnamate	0.0005	0.0228	↑2.4750
	DIMBOA	< 0.0001	0.0089	↓0.2167
	Equol	0.0016	0.0331	↑51.443
	Gluconate	0.0022	0.0347	↑7.674
	Vanillin	0.0048	0.0499	↑2.3458
Xanthine metabolism	1-Methylurate	0.0017	0.0342	↓0.3717

↓ indicated downregulation and ↑ indicates upregulation compared with findings in healthy control cats. Fold change was calculated for cats with CE relative to healthy cats.

**Figure 1:**
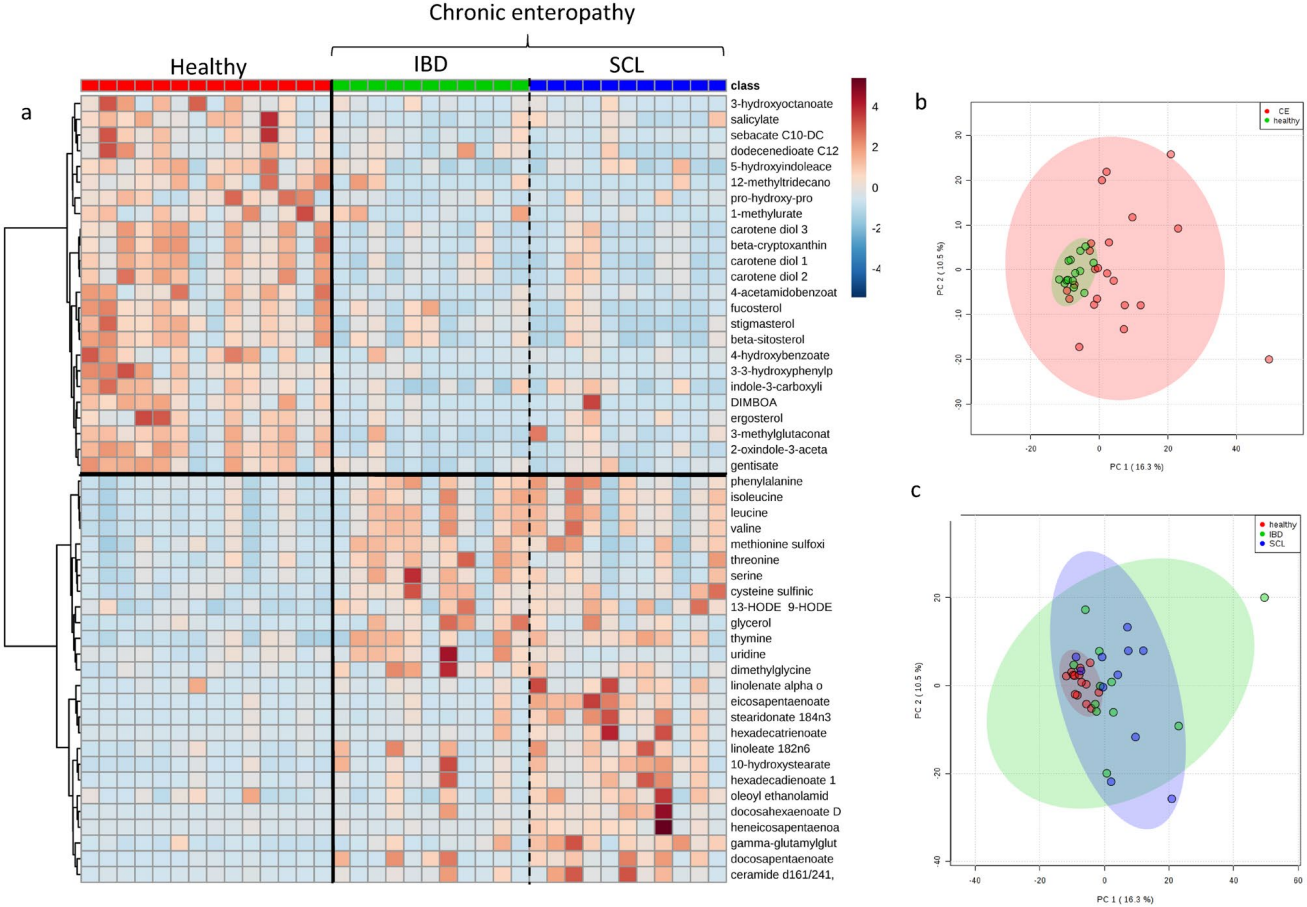
Multivariate analysis of the fecal metabolome of healthy cats and cats with chronic enteropathy. (**a**) Heat map showing metabolites that were significantly different between healthy cats and cats with inflammatory bowel disease (IBD) and alimentary small cell lymphoma (SCL). Groups are represented by the colored bars at the top of the figure as red (healthy, n = 14), green (IBD, n = 11), and blue (SCL, n = 11). Clusters can be identified between healthy cats and cats with chronic enteropathy (CE) but not between the disease subgroups IBD and SCL. (**b**) PCA score plots of metabolites in feces from healthy cats (green) and cats with chronic enteropathy (CE, red). (**c**) PCA score plots of metabolites in feces from healthy cats (red), cats with inflammatory bowel disease (blue), and cats with alimentary small cell lymphoma (green). A cluster can be identified for healthy cats but no specific clusters can be seen for the subgroups of IBD and SCL. Data was mean centered and divided by the standard deviation of each variable (autoscaled).

**Figure 2:**
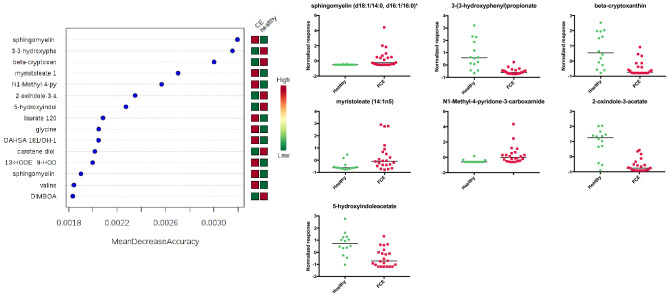
Random Forest variable importance plot and scatter plots of the top seven metabolites with the highest importance for the model accuracy. (**a**) Random Forest variable importance plot. Random Forest is a supervised, machine learning algorithm used for regression (prediction) and classification analysis of large data sets. This Random Forest algorithm was based on the comparison of cats with chronic enteropathy (FCE) and healthy control cats. The model identifies features with the highest predictive accuracy for health status. The variable importance plots shows the significant features identified by Random Forest. The features are ranked by the mean decrease in classification accuracy when they are permuted. A higher value indicates the importance of that metabolite in predicting the group (healthy vs. chronic enteropathy). Data was mean centered and divided by the standard deviation of each variable (autoscaled). (**b**) Scatterplots of the top seven metabolites from (**a**).

Principal component analysis (PCA), hierarchical clustering and a heatmap showing compounds that differed significantly between control cats and cats with CE indicated clustering of the two cohorts (Fig. 1a,b). Random forest classification revealed a group prediction, with a 16.7% out of bag (OOB) error rate (Table 3). The random forest importance plot identified 7 metabolites key in classifying the data with sphingomyelin (d18:1/14:0, d16:1/16:0), 3-(3-hydroxyphenyl)propionate, beta-cryptoxanthin, myristoleate (14:1n5), N1-methyl-4-pyridone-3-carboxamide, 2-oxindole-3-acetate, and 5-hydroxyindoleacetate, having the most influence on classification (Fig. 2).

**Table 3:** Random forest classification into healthy cats or cats with feline chronic enteropathy (FCE).

Actual group	Predicted group		Class error
	CE	Healthy	
FCE	21	1	0.05
Healthy	5	9	0.36

Overall out of bag (OOB) error rate is 16.7%.

Amongst biochemicals found to be increased in feces from cats with CE were various amino acids and their metabolites (e.g., aspartate, cysteine sulfinic acid, phenylalanine, leucine, and valine), fatty acids (e.g., arachidonate and eicosanoids), metabolites within vitamin E metabolism, and simple sphingolipids (e.g., ceramide, sphingomyelins, and sphingosines). Compounds significantly less abundant in cats with CE than in healthy controls included those involved in the tryptophan (i.e., indole-derivates) and vitamin A metabolism (i.e., carotendiols), and sterols.

Upon analysis of the CE subgroups, IBD and SCL, multivariate analysis revealed no visible clustering between cats with IBD and cats with SCL (Fig. 1a,c). Random forest analysis revealed poor group prediction with an OOB of 47.2% and class error rates of 82% and 55% for cats with IBD and cats with SCL, respectively. However, univariate analysis revealed 18 metabolites to differ significantly between healthy cats, cats with IBD, and cats with SCL. Post hoc analysis showed that 3 polyunsaturated fatty acids (i.e., eicosapentaenoate, heneicosapentaenoate, and stearidonate) within the eicosanoid family differed between cats with IBD and cats with SCL (Table 4).

**Table 4:** Significance of metabolites for the discrimination between the three groups, healthy controls, cats with inflammatory bowel disease (IBD), and cats with alimentary small cell lymphoma (SCL).

Subpathway	Metabolite	P value^a^	Q value	Multiple comparison test^b^		
				Healthy vs IBD	Healthy vs. SCL	IBD vs. SCL
**Amino acids**						
Glutamine	Gamma-glutamylglutamine	0.0004	0.0307	ns	SCL > H^e^	ns
Tryptophan	2-oxindole-3-acetate	0.0002	0.0282	H > IBD^e^	H > SCL^d^	ns
**Cofactors and vitamins**						
Nicotinate and nicotinamide metabolism	N1-Methyl-4-pyridone-3-carboxamide	0.0003	0.0307	IBD > H^c^	SCL > H^e^	ns
**Lipid**						
Fatty acid, hydroxyl	LAHSA (18:2/OH-18:0)	0.0004	0.0307	ns	SCL > H^e^	ns
	OAHSA (18:1/OH-18:0)	0.0004	0.0307	ns	SCL > H^e^	ns
	PAHSA (16:0/OH-18:0)	0.0006	0.0345	ns	SCL > H^e^	ns
Fatty acid, monohydroxy	10-hydroxystearate	0.0002	0.0282	IBD > H^c^	SCL > H^e^	ns
Polyunsaturated fatty acid	Heneicosapentaenoate (21:5n3)	0.0002	0.0282	ns	SCL > H^e^	SCL > IBD^c^
	Stearidonate (18:4n3)	0.0004	0.0307	ns	SCL > H^e^	SCL > IBD^d^
	Eicosapentaenoate (EPA; 20:5n3)	0.0006	0.0345	ns	SCL > H^e^	SCL > IBD^c^
	Hexadecatrienoate (16:3n3)	0.0006	0.0345	ns	SCL > H^e^	ns
	Nisinate (24:6n3)	0.0008	0.0394	ns	SCL > H^e^	ns
Sphingomyelins	Sphingomyelin (d17:1/16:0, d18:1/15:0, d16:1/17:0)	0.0002	0.0282	IBD > H^c^	SCL > H^e^	ns
Sphingomyelins	Sphingomyelin (d18:1/14:0, d16:1/16:0)	0.0005	0.0307	ns	SCL > H^e^	ns
Sterol	Stigmasterol	0.0001	0.0282	H > IBD^c^	H > SCL^e^	ns
**Xenobiotics**						
Benzoate metabolism	3-(3-hydroxyphenyl)propionate	0.0002	0.0282	H > IBD^d^	H > SCL^e^	ns
Chemical	4-acetamidobenzoate	0.0009	0.0435	H > IBD^e^	ns	ns
Food component/plant	DIMBOA	0.0002	0.0282	H > IBD^e^	H > SCL^d^	ns

*n*s not significant.

^a^Kruskal-Wallis test, three groups.

^b^Dunn’s post hoc.

^c^0.01 < P < 0.05.

^d^0.001 < P < 0.01.

^e^P < 0.001.

## Discussion

Our study revealed global metabolic changes in cats with chronic enteropathy as compared with healthy controls, with many metabolic pathways affected. Both hierarchical cluster analysis and PCA demonstrated clustering among cats diagnosed with chronic enteropathy and healthy control cats. Random forest analysis revealed a good class prediction of 80.6%. Univariate analysis showed a total of 84 metabolites to differ significantly between the two groups after controlling for a 5% FDR. Similar studies in humans with inflammatory bowel disease have also shown significant metabolomic differences between affected patients and healthy controls^10–12,30^. Although IBD in humans and CE in cats share only a few characteristics, the use of metabolomics data in humans with chronic enteropathies would support this type of approach in further assessing cats with various forms of CE. In addition, metabolic consequences of feline CE and human IBD appear to be very similar. Metabolites or metabolite families commonly found to be affected by IBD in humans are amino acids^8,10–13,30^, bile acids^10^, fatty acids^10,11^, and metabolites of the tryptophan pathway^10,31^.

**Figure 3:**
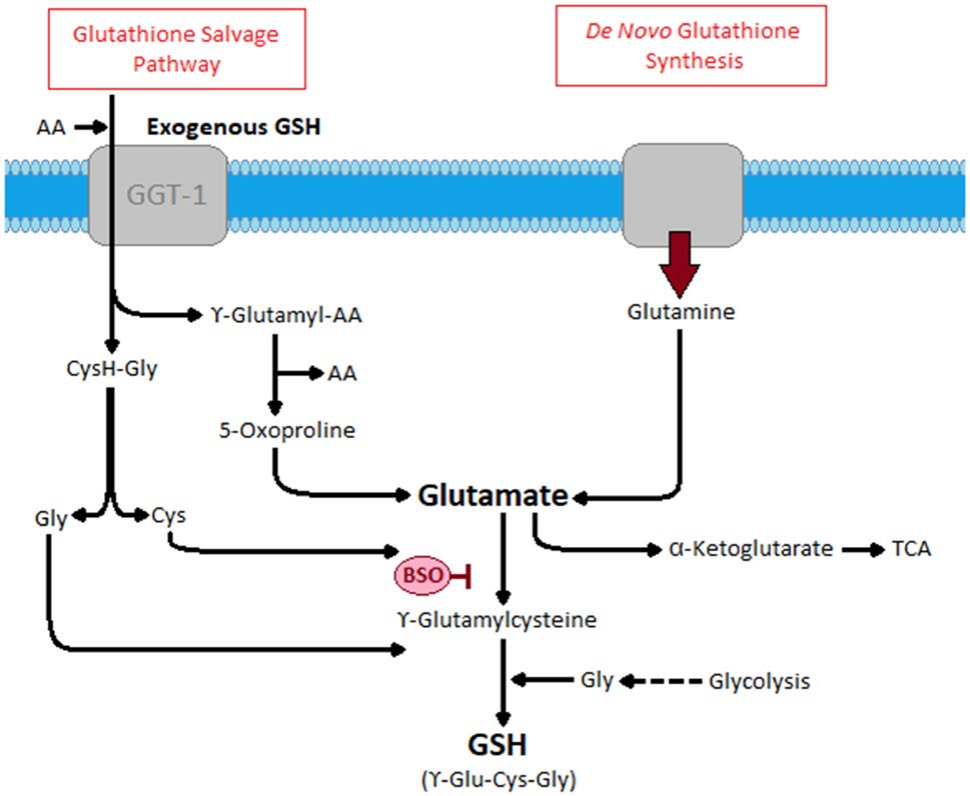
Glutathione salvage pathway. This figure details the metabolism of gamma-glutamyl amino acids to glutathione, a major cellular defense mechanism of oxidative stress.

We found multiple amino acids to be increased in the feces from cats with CE, indicating malabsorption, likely as consequence of mucosal inflammation, neoplastic infiltration, or both. Interestingly, 2-hydroxybutyrate/2-hydroxyisobutyrate and gamma-glutamylglutamine, two metabolites related to the glutathione-metabolism, were found to be significantly more abundant in feces from cats with CE based on both univariate and multivariate analysis. Gamma-glutamyl amino acids are precursors for the formation of glutathione in the gamma-glutamyl cycle (glutathione salvage pathway)^32^ (Fig. 3). Reactive oxygen species have been implicated to contribute to tissue injury in patients with Crohn’s disease and ulcerative colits^33,34^. Glutathione is the major intracellular antioxidant and thus a critical part of the defense mechanism against oxidative stress in inflammatory conditions such as CE (Fig. 3)^35,36^. Consequently, glutathione precursors are in high demand during catabolic conditions and corresponding higher loads of oxidative stress^30^. Increased fecal concentrations of gamma-glutamyl glutamine in cats with CE might indicate increased loss and correspond to a decreased mucosal availability and glutathione synthesis.

Our study showed arachidonate to be more abundant in feces from cats with CE. Arachidonate is a well described mediator of inflammation, including intestinal inflammation, and a precursor for prostaglandins, which are essential immune signaling molecules^37^. In addition, arachidonate has been found to increase the expression of intercellular adhesion molecule 1 (ICAM-1)^38^. Adhesion molecules such as ICAM-1 and mucosal addressin cell adhesion molecule (MAdCAM) are involved in recruiting leucocytes to the site of inflammation and have been identified as therapeutic targets for human patients with IBD^39^. Increased fecal arachidonate may reflect mucosal upregulation during inflammation and subsequent leakage into the fecal stream. On the other hand, omega-3 polyunsaturated fatty acids, including eicosapentaenoate, have been found to be significantly more abundant in the SCL subgroup of cats with CE compared to healthy controls.

Eicosapentanoate and other omega-3 fatty acids have anti-inflammatory properties and inhibit conversion of arachidonate into the pro-inflammatory thromboxane-2 and prostaglandin-2 families^40^. Increased fecal levels of omega-3 fatty acids might indicate an increased loss or malabsorption and thus may correspond to the increased arachidonate concentration found in this study.

We also found several metabolites of the indole family and pathway to be altered in cats with CE. Amongst a wide range of structurally divergent exo- and endogenic chemicals^41^, indole derivates are important ligands for the aryl hydrocarbon receptor (AhR)^42^. AhR signaling participates integrally in intestinal mucosal homeostasis by acting on innate and adaptive immune cells as well as on epithelial renewal and mucosal barrier function^43^. Tryptophan plays a central role in AhR activation because it is transformed into indole and indole derivates by the gut microbiota. Studies in murine and porcine models of colitis have found significantly reduced disease activities after oral supplementation with indole-3-propionate or L-tryptophan by activating anti-inflammatory pathways mediated by IL-10 and IL-22^42,44^. We found indole derivates, such as 2-oxindole-3-acetate and 5-hydroxyindoleacetate, to be significantly less abundant in feces from cats with CE. Although, tryptophan did not significantly differ between CE and control cats, it showed a trend (p = 0.0855, q = 0.2385) towards a higher fecal excretion in cats with CE^42,45,46^ (Appendix C, Tables 5.5, 5.6). These findings might indicate disrupted transformation of tryptophan into indole derivates, possibly linked to intestinal dysbiosis in this cohort of cats with CE.

Finally, our study showed increased concentrations of several simple sphingolipids in feces from cats with CE. The role of sphingolipids in IBD is complex and incompletely understood. It appears, however, that a functional equilibrium between simple sphingolipids (e.g., sphingomyelin, sphingosine, ceramide) and complex sphingolipids (e.g., gangliosides GM3 and GD3) is essential in maintaining intestinal homeostasis^47^. Proinflammatory sphingolipid patterns have previously been described, with increased concentrations of sphingomyelin and ceramide in feces of animal models of colitis^48,49^ as well as in the ileal mucus of human patients with Crohn’s disease^50^ indicating that sphingomyelin and ceramide accompany and possibly aggravate chronic intestinal inflammation.

Fecal extracts obtained from cats with IBD and SCL revealed similar global changes in metabolic profiles. However, changes observed in cats with IBD were less pronounced than those observed in cats with SCL. This suggests that metabolic consequences are more severe in SCL than in IBD. In particular, higher concentrations of polyunsaturated fatty acids of the eicosanoid family were found in SCL.

This study had several limitations. A clinical diagnosis of IBD was based on the presence of chronic gastrointestinal signs of at least 3 weeks duration, the absence of known enteropathogens or other causes of signs of gastrointestinal disease, and the histopathologic confirmation of intestinal inflammation^51^. Hence, we did not exclude food-responsive enteropathy in all participating cats. However, in response to dietary interventions human IBD patients can show complete or partial resolution of clinical signs without being reclassified as having food-responsive enteropathy^52–55^. Fecal cultures for specific pathogens were not performed. However, gastrointestinal disease caused by bacteria usually cause acute diarrhea. In addition, all cats showed either lymphoplasmacytic enteritis or lymphoma on histopathologic examination. Cats with neutrophilic enteritis, a hallmark of gastrointestinal bacterial infections, were excluded from the study. Differentiating IBD from SCL can impose significant challenges with clinical signs and histopathologic changes commonly overlapping^3^. Moreover, mucosal changes may have an irregularly distribution, inflammatory and neoplastic lesions often coincide and inflammatory lesions might even progress to lymphoma over time^3,4^. Therefore, using currently available measures, the classification into groups of IBD and SCL will always be associated with a degree of ambiguity. However, in all cats intestinal biopsies were available and results of immunohistochemistry and clonality testing were utilized, where indicated, to provide the highest accuracy in category assignment.

Inter-individual variation of metabolomic profiles have been described in humans, dogs, and cats and might be related to a variety of exogenous and endogenous factors such as the environment, diet, gut microbiota, xenobiotics, and the genome^14,17,56^. This study intended to identify clinically relevant perturbations of the fecal metabolome of cats with CE compared to healthy subjects. Demographic characteristics like age, sex, and breed were controlled for in this study. However, we did not control for environmental factors, such as diet and housing, since this likely would have impacted the clinical relevance of our results. This concept is supported by recent studies showed that standardization in is a major source of poor reproducibility preclinical trials^57,58^. Although, we cannot exclude that these factors confounded our results, most are in line with findings across different species with spontaneous or induced IBD and thus likely reflect true changes of the fecal metabolome. In addition, most cats in both groups were housed indoors and fed a variety of different commercial diets. Another limitation is the limited number of animals in this study. However, untargeted “omics” analyses usually provide only relative changes between subjects or groups and are generally conducted using a smaller sample size. Therefore, all untargeted analyses have to be followed by targeted assays to confirm results of those fishing expeditions on a larger number of subjects. However, this was beyond the scope of this study.

In summary, our study revealed global metabolic changes in cats with CE compared to healthy controls. Many metabolic pathways were affected such as amino acids, fatty acids (e.g., arachidonic acid and eicosanoids), sphingolipids, and metabolites of the tryptophan pathway (i.e., indole-derivates). Metabolic profiles resembled patterns found in humans and other animal models with IBD and thus metabolic consequences of feline CE and human IBD might be similar. Future studies of the mucosal and serum metabolome of cats with CE should be conducted to further elucidate the origin of metabolic perturbations and allow further insights into pathogenesis. In addition, targeted analysis of the compounds found to be altered in cats with CE is indicated to confirm results and to investigate their value as non-invasive biomarkers.

## Materials and methods

### Study approval and enrollment

This prospective study was conducted at the Veterinary Medical Teaching Hospital at Texas A&M University between May 2015 and September 2017. The study protocol was approved by the Texas A&M University Institutional Animal Care and Use Committee (IACUC 2015-0276 CA and IACUC 2014-0369 CA). All experiments were performed in accordance with relevant guidelines and regulations. Cat owners provided written informed consent prior to study enrollment. Cats with clinical signs of chronic enteropathy (n = 22) and control cats (n = 14) were recruited from the hospital population at the Small Animal Hospital of Texas A&M University in College Station, Texas or the Veterinary Specialty Hospital in San Diego, California^59^.

The health status of cats in the group considered healthy was verified by an owner questionnaire on general and gastrointestinal health^59^. The questionnaire covered the following areas: attitude/activity, appetite, drinking, urination, chronic illnesses, weight loss, vomiting, diarrhea, and treatment with antibiotics, antacids, anti-inflammatory drugs, or steroids^59^. In addition, a physical examination and blood testing was available and performed by a single board-certified internist (SM) in 11 of 14 cats. The body condition score was assessed using a previously established a nine-point condition scoring system^28^. Blood was collected from a peripheral vein or the jugular vein and the following tests were performed: complete blood count, serum chemistry profile, total T4, cobalamin, folate, feline pancreatic lipase immunoreactivity (fPLI), and feline trypsin-like immunoreactivity (fTLI). Cats with gastrointestinal signs (weight loss, hyporexia, vomiting > 2×/ month, diarrhea) within 6 months prior to enrollment were excluded. In addition, cats with systemic diseases, chronic illnesses or clinically significant laboratory abnormalities were excluded from the study^59^. Finally, cats that had received any antibiotics, antacids, anti-inflammatory drugs, or corticosteroids within the past 6 months were excluded^59^.

Cats with clinical signs of chronic enteropathy (weight loss, hyporexia, vomiting, diarrhea) of at least 3 weeks duration were eligible for enrollment into the group of cats with chronic enteropathy^59^. Extra-gastrointestinal disease as well and, where indicated, infectious intestinal diseases were excluded based on a complete blood count, serum chemistry profile, total T4 and fecal flotation^59^. All cats in this group underwent gastro-duodenoscopy and ileo-colonoscopy for diagnostic purposes. Histopathologic examination of H&E stained endoscopic formalin-fixed, paraffin-embedded (FFPE) tissue sections was performed by board-certified pathologists (MA or JSE) blinded to the clinical status of the cats^59^.

Cases with a histopathological diagnosis of SCL or cases where the pathologist was suspicious of an underlying SCL underwent additional diagnostic testing with immunohistochemistry and PCR for antigen receptor rearrangement testing for diagnostic confirmation. A final diagnosis of IBD or SCL was reached upon integration of results from histopathology, immunohistochemistry, and PARR based on the current EuroClonality/BIOMED-2 guidelines for interpretation and reporting of Ig/TCR clonality testing in suspected lymphoproliferations^59–62^. Cats that had received antibiotics within 4 weeks or corticosteroids within the past 2 weeks prior to fecal sampling were excluded from the study^59^.

### Sample collection

Fecal samples were collected after spontaneous defecation or digitally while the cat was under anesthesia and prepared for endoscopy. Fecal samples were refrigerated or frozen immediately after collection and shipped to the Gastrointestinal Laboratory at Texas A&M University on ice packs or dry ice within 24 h. Upon arrival, fecal samples were immediately divided into alliquots and stored at − 80 °C until analysis^59^.

### Metabolite extraction

Untargeted fecal metabolomic analysis was performed by Metabolon, Inc. (Durham, NC) as previously described^63–65^. Metabolites were extracted from lyophilized and homogenized samples using methanol extraction. Extracts were analyzed by an ACQUITY ultra-performance liquid chromatographer (Waters, Milford, CA) and a ThermoFisher Scientific Q-Exactive high resolution mass spectrometer interfaced with a heated electrospray ionization (HESI-II) source and Orbitrap mass analyzer (ThermoFisher Scientific, Waltham, Massachusetts) operated at 35,000 mass resolution. The scan range covered 70–1000 m/z. Metabolite identification was performed by automated comparison of the ion features in the experimental samples to a reference library. Chemical standard entries included retention time, molecular weight (m/z), preferred adducts, and in-source fragments as well as associated MS spectra, and were curated by visual inspection for quality control using software developed at Metabolon. Peaks were quantified using the area-under-the-curve. Compounds were corrected for inter-day variation by registering the medians to equal 1.00 and normalizing each data point proportionally. Missing values were imputed with the observed minimum for each compound.

### Statistical analyses

Patient demographics were compared by the Mann–Whitney or Fisher’s exact test as appropriate. Differences in the abundance of fecal metabolites between control cats and cats with CE were evaluated using a Mann–Whitney test. A post hoc analysis to assess differences in the abundance of metabolites between the subgroups (i.e., controls, IBD, SCL) was performed by Dunn’s test. Statistical significance was set at p < 0.05. Results were adjusted by False Discovery Rate (FDR) and reported as the q-value where appropriate. Univariate analysis was performed using Prism 7.0b (Graph Pad Software, La Jolla, CA) and JMP Pro 14.1.0 (SAS Institute Inc., Cary, NC).

Multivariate analysis was performed using MetaboAnalyst^66^. Data was mean centered and divided by the standard deviation of each variable. PCA and hierarchical clustering was performed and a heatmap was created as a visual aid for the dendrogram. Random forest regression analysis was used to evaluate the classification performance of metabolomics.

### Ethical approval

This article does not contain any studies with human participants performed by any of the authors.

### Research involving animal rights

All applicable international, national, and/or institutional guidelines for the care and use of animals were followed.

## Supplementary Information

Supplementary Information

## Data Availability

The datasets generated and/or analyzed during the current study are available from the corresponding author on reasonable request.
